# A formalin-inactivated immunogen against viral encephalopathy and retinopathy (VER) disease in European sea bass (*Dicentrarchus labrax*): immunological and protection effects

**DOI:** 10.1186/s13567-016-0376-3

**Published:** 2016-09-02

**Authors:** Noelia Nuñez-Ortiz, Francesco Pascoli, Simona Picchietti, Francesco Buonocore, Chiara Bernini, Marica Toson, Giuseppe Scapigliati, Anna Toffan

**Affiliations:** 1 Department for Innovation in Biological, Agro-food and Forest Systems, University of Tuscia, Viterbo, Italy; 2 Centro di Referenza Nazionale (NRL) per le patologie dei pesci, molluschi e crostacei, Istituto Zooprofilattico Sperimentale delle Venezie, Legnaro (PD), Italy; 3 Epidemiologia applicata agli animali acquatici, Istituto Zooprofilattico Sperimentale delle Venezie, Legnaro (PD), Italy

## Abstract

The European sea bass (*Dicentrarchus labrax*) is an important farmed fish species in the Mediterranean area, very sensitive to the infection by encephalopathy and retinopathy virus (VERv), or *Betanodavirus*, which causes massive mortalities. Effective vaccines to fight the pathology are not yet available and in this work we describe a promising intraperitoneal immunization route against VERv of sea bass juveniles. We performed intraperitoneal and immersion immunization trials with a VERv (isolate 283.2009 RGNNV) inactivated by formalin, β-propiolactone and heat treatment. Interestingly, the intraperitoneal immunization with formalin-inactivated VERv induced a significant antigen-specific IgM production, differently from other inactivation protocols. However, the same formalin-inactivated antigen resulted in very low IgM antibodies when administered by immersion. Following the intraperitoneal injection with formalin-inactivated virus, the quantitative expression of the antiviral MxA gene showed a modulation of transcripts in the gut after 48 h and on head kidney after 24 h, whereas ISG12 gene was significantly up-regulated after 48 h on both tissues. In immersion immunization with formalin-inactivated VERv, a modulation of MxA and ISG12 genes after 24 h post-treatment was detected in the gills. An effective uptake of VERv particles in the gills was confirmed by immunohistochemistry using anti-VERv antibodies. Lastly, in challenge experiments using live VERv after intraperitoneal immunization with formalin-inactivated VERv, we observed a significant increase (81.9%) in relative survival percentage with respect to non-immunized fish, whereas immersion immunization resulted in no protection. Our results suggest that intraperitoneal immunization with formalin-inactivated VERv could be a safe and effective strategy to fight *Betanodavirus* infection in European sea bass.

**Electronic supplementary material:**

The online version of this article (doi:10.1186/s13567-016-0376-3) contains supplementary material, which is available to authorized users.

## Introduction

Viral encephalopathy and retinopathy (VER), also known as viral nervous necrosis (VNN), is a severe infectious disease characterized by neuropathological changes and associated with high mortality in several fish species.

Viruses belonging to the *Nodaviridae* family, genus *Betanodavirus*, are non-enveloped single strand positive RNA virus characterized by an extremely high resistance to chemical and physical agents [1]. *Betanodavirus* is a highly pathogenic virus able to evade the host’s protective systems and can either replicate and transmit progeny to other cells or remain in a latent condition [2]. *Betanodavirus* is one of the most significant viral pathogens of finfish and represents a bottleneck for development of mariculture in several countries [3, 4].

Several experimental vaccines against VNN have been tested so far [5–7]. However, most of them have been tested on grouper (*Epinephelus* spp.), very likely the most important and valuable fish amongst the VER-susceptible species [8]. An inactivated VERv vaccine (RGNNV genotype) against VER of sevenband grouper (*Epinephelus septemfasciatus*) has been produced and marketed in Japan. Sea bass (*Dicentrarchus labrax*) is the second most farmed species in the Mediterranean Sea (FEAP 2005–2014) and also highly susceptible to VER. Notably, the VER introduction in the Mediterranean basin dates back to the early 90s and was firstly detected in sea bass hatcheries [9]. All stages of sea bass are highly sensitive to *Betanodavirus* but mortality can vary depending on the fish age and water temperature [10]. Larval and juvenile stages are commonly the most affected, reaching mortalities up to 100%. Infected larvae and juvenile stages often show flexing of the body, muscle tremors and abnormal swimming behaviour, which includes vertical positioning and spinning resulting from traumatic lesions [11]. *Betanodavirus* also causes hyperinflation of the swim bladder, with diseased fish present primarily at the surface. In adults, where the mortaly rate can reach 50–60% [10, 12], the most common clinical sign is abnormal swimming.

Despite the huge losses to Mediterranean aquaculture, in particular to sea bass farms, very few vaccines have been tested in this species. Only few experimental products based on recombinant protein or synthetic peptides have been used by injecting intramuscularly juveniles sea bass of 20–66 g [13, 14]. The use of a recombinant capsid protein has given interesting results but, at present, a vaccine against *Betanodavirus* for *D. labrax* is commercially not available.

The objectives of the present work were: (1) to investigate the best inactivation system for the production of immunogens, (2) to test the efficacy of the best inactivation method to protect experimentally, infected fish, and (3) to evaluate the immune response of immunized European sea bass. To achieve this we used two size-classes of fish, on the base of previous studies from our laboratory showing that intraperitoneal injection is inadvisable in fish with a mean weight lower than 5 g, and that bath challenge is not applicable over that weight.

## Materials and methods

### Fish

Eight hundred and twenty (820) juvenile European sea bass (410 with an average weight of 2.10 ± 0.25 g and 410 with an average weight of 6.30 ± 0.45 g) were transferred from a VER-free commercial farm (previously tested by PCR to be VERv-free, data not shown) to the experimental aquarium at the Istituto Zooprofilattico Sperimentale delle Venezie (IZSVe, Legnaro-PD, Italy). On arrival, forty fish were sampled in order to be checked for the most common pathogens (parasites, bacteria and viruses). Preliminary analyses confirmed that all the fish were pathogen-free. After this first phase, they were equally distributed in 9 different close system tanks according to their size (4 tanks with 80 fish of bigger size, and 5 tanks with 80 fish of smaller size), with 300 L of artificial salt water at 25‰ of salinity, at a temperature of 22 ± 1 °C, oxygen 6 ± 0.5 ppm and artificial photoperiod of 8 h of light and 16 h of darkness.

### Virus

The virus was selected according to its degree of high pathogenicity for fish. In 2009, RGNNV 283.2009 was isolated from severely affected sea bass during an outbreak in a commercial farm in the northern Adriatic Sea. The virus had been previously used in the IZSVe experimental aquarium, showing a mortality rate of >35% by immersion exposure in sea bass [15, 16]. The isolate was propagated on E-11 cells (10 passages) [17], a clone of SSN-1 cell line [18], in 150 cm^2^ tissue culture flasks using L-15 medium (Leibovitz) (Sigma-Aldrich) without fetal calf serum. The collected virus was subjected to titration by endpoint dilutions assays. Titres were calculated according to the Spearman–Karber formula [19] and expressed as TCID_50_/mL.

### Immunogens preparation

The E-11 cell medium containing VERv was centrifuged at 2000 *g* for 10 min to remove cell debris, and then, the virus was inactivated with three different methods to prepare three different immunogens (titre 6.31 × 10^7^ TCID_50_/mL). Formalin inactivation was carried out by adding buffered formalin (Carlo Erba, Italy) at a final concentration of 10 µL/mL and left at room temperature (22–25 °C) for 1 week. The β-propiolactone (BPL, Ferak, Berlin) inactivation was performed by adding 20 µL/mL of BPL for 3 h at 37 °C. Inactivation by heat treatment was done in a heated bath at 70 °C for 1 h. All immunogen preparations were checked after treatment using virological analyses according to a standard procedure [16], performing three sequential serial blind passages to ensure the complete inactivation of the pathogen. One batch of virus was kept untreated and used for a live virus immersion trial.

### Fish immunization and challenge

After a period of acclimation of 10 days, fish were immunized as follows. Bigger fish (average weight of 6.30 ± 0.45 g) were sedated with 10 mg/L tricaine methasulfonate (MS-222) (Sigma-Aldrich) and then immunized by intraperitoneal injection (i.p.) of 0.1 mL/fish (using syringes with a 26-gauge needle) of the three different immunogens preparations described above (one per tank); whereas smaller fish (average weight of 2.10 ± 0.25 g) were immunized by immersion in a 30-L water tank with aerator for 2 min with a final titre of 10^6^ TCID_50_/mL of the same inactivated formulations. A new group was added and immersed with a sub-optimal dose of live virus (approximately 10^4^ TCID_50_/mL), following a previous protocol [20]. The controls for experimental groups were done by i.p. injection and immersion (mock-immunization) with 0.01 M PBS (Sigma-Aldrich). These controls acted as negative control for immunological assays and as positive control in the challenge. One tank was left untreated as negative control, in order to assess general viability during treatments and challenge. Thirty days post immunization, blood and brain samples were collected from fish (*n* = 10) from all groups.

According to the results from immunological analyses (see below), we focused our study on formalin-inactivated VERv groups, from which we performed real-time quantitative PCR (qPCR) to measure the transcription of antiviral genes. We then tested the survival rate in the same groups after challenge with live virus.

After 30 days post immunization (dpv), the formylated groups were challenged with RGNNV 283.2009 by immersion or intramuscular injection, according to the prior immunization route. As for immersion, each group of 50 fish was transferred to a 30-L water tank with aerator and infected for 4 h by adding 30 mL of virus solution (final titre 10^6^ TCID_50_/mL) to the water. For intramuscular injection, 50 fish were injected (after anesthesia) with 0.1 mL of virus solution (approximately 6.31 10^6^ TCID_50_/fish). The “positive control” group, mock-immunized by i.p. and immersion respectively by using PBS 0.01 M, was infected by immersion or injected as explained before. The “negative control” group was either mock infected by immersion by adding minimum essential medium (MEM-10) without virus to the water, or injected with 0.1 MEM-10 rather than with the virus solution administered to the intramuscularly infected group.

Fish were then transferred back to the original tanks and kept at a temperature of 25 ± 1 °C, oxygen 6 ± 0.5 ppm and artificial photoperiod of 8 h of light and 16 h of darkness. Fish were checked twice a day and the dead ones were removed. Brain samples were collected individually from each dead fish and stored at −80 °C. The experiment ended on day 30 and the relative percent survival (RPS) calculated. All the remaining fish were euthanized by an overdose of MS-222, sampled and stored at −80 °C until analyses.

### Immunological analyses

The blood from juveniles sea bass was collected from the caudal vein of lethally anaesthetized fish (overdose of MS-222). Sera were obtained by centrifugation at 1000 *g* for 5 min.

#### Indirect ELISA

VERv-specific IgM detection was performed using a previously developed Indirect ELISA [20]. The ELISA assays data are presented as the mean absorbance ± SD. Ten single samples by group were measured in duplicate wells and optical density values (OD 450 nm) of control wells were automatically subtracted from samples values.

#### Serum neutralization tests

Serial twofold dilutions (1:20–1:2560) of heat inactivated serum were prepared in a 96 well plate (Corning) with MEM Leibovitz medium without FBS. Diluted sera were incubated with a defined amount of infectious virus (100 TCID_50_/50 µL), 4 wells were used for each sample. After incubation overnight at +4 °C, the virus-serum mixture was added to a confluent E-11 cell line and incubated for 10 days at 25 °C. Plates were observed every 3 days for appearance of cytopathic effects (CPE). The neutralization value of a virus is defined as the reciprocal of the highest dilution of serum that completely inhibits CPE.

#### Real-time quantitative PCR

At 0, 24 and 48 h post immersion with formylated virus, 4 pools of 5 gill archs per time points per group were collected, whereas after i.p. immunization with formylated virus, 3 single samples of head kidney and gut were collected per time points and per group. Three muscle samples from control groups were sampled behind the pectoral fin. Samples were immediately placed in 1 mL of denaturing solution (Tripure, Roche), total RNA was isolated following the manufacturer’s instructions and subsequently suspended in DEPC-treated water. For reverse transcription, the BioScript RNase H minus (Bioline) enzyme was used following the manufacturer’s instructions. The absence of DNA contamination was checked using sea bass β-actin primers that bracketed an intron [20]. The transcripts relative to MxA and ISG12 genes were determined with an Mx3000P™ Real-time quantitative PCR system (Stratagene) equipped with version 2.02 software, using the Brilliant SYBR Green Q-PCR Master Mix (Stratagene) following the manufacturer’s instructions. ROX was used as internal passive reference dye. Specific PCR primers (Table 1) were designed for the amplification of products (ca. 200 bp) from the conserved region of all the analyzed genes and ribosomal 18S rRNA was used as house-keeping gene. A 10 ng of cDNA template was used in each PCR reaction. PCR conditions were as follows: 95 °C for 10 min, followed by 35 cycles of 95 °C for 45 s, 52 °C for 45 s and 72 °C for 45 s. Reactions were performed in triplicate for each template cDNA, which was replaced with water in all blank control reactions. Each run was terminated with a melting curve analysis, which resulted in a melting peak profile specific to the amplified target DNA. The analysis was carried out using the endpoint method option of the Mx3000P™ software, allowing the fluorescence data to be collected at the end of each extension stage of amplification. A relative quantification was performed by comparing the levels of the target transcript to the reference transcript (18S rRNA). Quantitative assessment was based on the determination of the threshold cycle (Ct), defined as the cycle at which a statistically significant increase in fluorescence above the background signal was detected using the ΔΔCt method. The calibrator, defined as 1.0 value, was the average of 3 muscle samples from control fish due to the low expression of these antiviral genes in the muscle.

**Table 1:** **Specific Real-time quantitative PCR primers**

Gene	Primer sequence	EMBL accession
18S rRNA	FW: 5′-CCAACGAGCTGCTGACC-3′ RW: 5′-CCGTTACCCGTGGTCC-3′	AY831388
ß-actin	FW: 5′-ATGTACGTTGCCATCC-3′ RW: 5′-GAGATGCCACGCTCTC-3′	AJ493428
MxA	FW: 5′-GTCTGGAGATCGCCTCT-3′ RW: 5′-GTGGATCCTGATGGAGA-3′	JN807551
ISG12	FW: 5′-CCTGGTACAGCTGCTGT-3′ RW: 5′-AGCTGCTCCTGCTGACT-3′	FN665389

### Virological analysis

#### Virus isolation in cell culture

Samples (cell culture supernatant and fish brains) were examined by previously described standard virological techniques [16].

Real Time RT-PCR analyses for detection of Betanodavirus RNA2 were performed using a slightly modified version of the published protocol [21]. Briefly, total RNA was extracted from 100 µL of the sample using the NucleoSpin^®^ RNA kit (Macherey-Nagel) and according to the manufacturer’s instructions. Real Time RT-PCR was conducted with RotorGene Q (Qiagen), Rotor Gene 6000 (Corbett, Australia) using the OneStep RT-PCR Kit (Qiagen) following the manufacturer’s recommendations, adding Random primers (Applied Biosystems) and RNase Inhibitor (Promega). Primers and probe were used at a final concentration of 0.9 and 0.75 µM, respectively, random primers at concentration 1X, RNase Inhibitor at final concentration of 8 U/µL and thermal cycling was performed at 50 °C for 30 min and denaturation at 95 °C for 10 min followed by 45 cycles of 95 °C for 10 s, 54 °C for 35 s and 72 °C for 1 s, with a final incubation at 40 °C for 30 s. Data analyses were performed with the Rotor Gene 6000/RotorGene Q series software.

### Immunohistochemistry

For IHC, gills from sea bass immersed with formalin-inactivated VERv were sampled at 48 h post-immersion (1 min). Gills were fixed in ice-cold Bouin’s fixative for 7 h in Bouin’s at 4 °C. After embedding in paraplast, blocks were serially sectioned at a thickness of 7 μm as previously described [22]. The immunohistochemical reaction was performed by ABC-peroxidase with nickel enhancement, as previously described [23]. Serial sections were incubated with the mAb 4C3 [24] (that recognizes VERv capsid protein) diluted 1:10 in PBS 0.1 M (Sigma-Aldrich), pH 7.3 containing 5% normal horse serum and 0.1% sodium azide, or with pAb 283 [25] (that recognizes the whole virus) diluted 1:3000 using PBS containing 5% normal goat serum and 0.1% sodium azide. Controls were carried out by omitting the primary antibody. Thereafter, sections were incubated for 60 min with biotinylated horse anti-mouse or anti rabbit IgG sera (Vector Labs., Burlingame, USA) diluted 1:1000 with PBS containing 0.1% sodium azide and 1% BSA, followed by incubation for 60 min with avidin-biotinylated peroxidase complex (ABC, Vectastain Elite, Vector). Following rinses and staining (diaminobenzidine and nickel enhancement), sections were dehydrated, mounted and examined under a Zeiss microscope equipped with a colour video camera (Axio Cam MRC, Arese, Milano, Italy) and a software package (KS 300 and AxioVision).

### Statistical analysis

The number of fish per experimental group was calculated according to the expected mortality rate (35%) and 0.05 as α error (one-side) and power 80% (1 − β = 0.80). Each fish in the study was followed over time and the event “death” was recorded and verified by Real Time RT-PCR.

The Kaplan–Meier method was used to estimate the survival function from lifetime data, which allowed to draw the survival curve for each group as a step curve and to measure the length of time the fish would survive the infection. In the graph, the y-axis plot indicates the cumulative probability of the surviving fish at each time [26]. To compare the different survival curves, the non-parametric Wilcoxon–Breslow–Gehan test was used for equality of survivor functions. One-sided tests with a *p* < 0.05 were considered as significant. Statistical analyses for potency test were performed using the STATA 12.1 software.

Statistical analysis for immunological parameters was performed by Graph Pad Prism 6.0 software statistical package. The data from two differents experiments are presented as the mean ± SD of four pools of five organs in the gills and three single samples in the head kidney and gut in Real-time quantitative PCR and ten single samples for Indirect ELISA. The statistical significance among groups for qPCR and Indirect ELISA was determined using One-way ANOVA followed by Bonferroni’s multiple comparison test and the level for accepted statistical significance was *p* < 0.05.

## Results

### Virological analysis

All three inactivation methods proved to inactivate *Betanodavirus* completely, with no viral growth in cell culture after 3 blind passages. The immunogens were then suitable to be used for experimental trials on animals.

No mortality was observed in fish both during and after immunization in all the treated groups. Thirty days post immunization, brains from immunized fish were collected and checked for VERv positivity by Real Time RT-PCR and virological analysis.

Positivity was found only in the live virus immersion immunized group (average ct 20.88 ± 4.29), whereas all the others gave negative results.

### Immunological analyses

Anti-VERv specific IgM was detected by Indirect ELISA in serum samples after immersion and intraperitoneal immunization with the virus inactivated by different ways.

**Figure 1:**
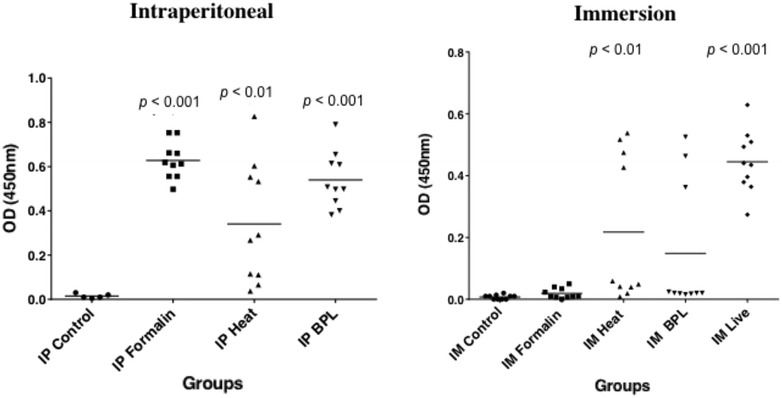
**Detection of VERv-specific IgM after immersion and intraperitoneal immunization with** ***Betanodavirus*** **inactivated by different methods.** Dilutions 1/60 of sera from different groups collected 30 days postimmunization were tested by Indirect ELISA using as antigen 10^6^ TCID_50_ per well of viral preparation and Pab IgM (diluted 1:10000). Background values (no viral preparation added) were automatically subtracted from OD 450 nm values of samples.

In all the samples injected intraperitoneally the presence of anti-VERv specific antibodies was detected (Figure 1). Fish immunized with formalin-inactivated VERv presented a high specific IgM production (OD 450 nm of ca. 0.627 ± 0.08) with respect to the control group; as well, fish immunized with BPL-inactivated VERv presented a high specific IgM production (OD 450 nm of ca. 0.540 ± 0.12), although a high standard deviation was observed in the latter case. Moreover, fish immunized with temperature-inactivated VERv presented a high standard deviation; some samples produced a high level of specific IgM while a lower but detectable IgM level was observed in another subjects (OD 450 nm of ca. 0.340 ± 0.272).

Indirect ELISA results referring to sea bass immersed in different preparations are also shown in Figure 1, where those exposed to a sub-optimal dose of live virus presented a quantity of specific IgM (OD 450 nm of ca. 0.445 ± 0.1) higher than that found in the formalin-inactivated VERv group, which has a lower antibody production. Temperature-inactivated VERv and BPL-inactivated VERv immunized groups both presented a higher standard deviation compared with the formalin-inactivated one due to the uneven responses among examined samples. As negative controls, samples from mock-immunized fish (intraperitoneally and by immersion) showed no detectable IgM antibody, as expected.

The serum neutralization test showed neutralizing antibodies against RGNNV 283.2009 only in the formalin-inactivated VERv i.p. immunized group (average titre GMT 3.8 ± 2.2), whereas all the others showed negative results (data not shown).

**Figure 2:**
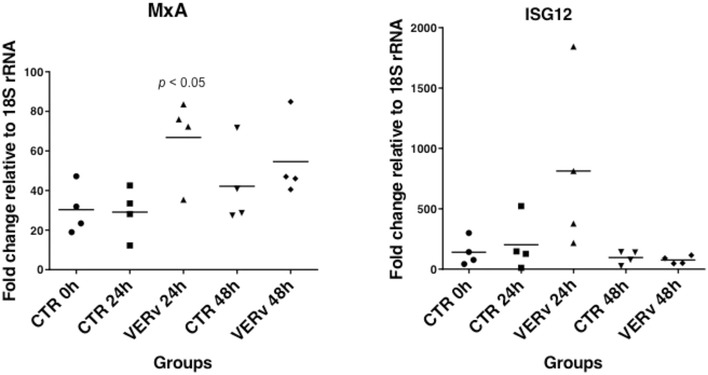
**Real-time quantitative PCR.** The expression level of genes coding for MxA and ISG12 in gills are shown in control fish (CTR Group) and in immersed immunized fish (VERv Group) at 24 and 48 h post immersion with formalin-inactivated virus. The quantitative PCR amplification was performed in PCR arrays, and each point represents the mean ± SD of four diverse pools with 5 fish each.

Quantitative-PCR, after immersion and intraperitoneal immunization with formalin-inactivated VERv, was performed to analyze the expression of antiviral immune response genes in the gills, head kidney and gut. Transcripts levels of MxA and ISG12 genes in gills after immersion are shown in Figure 2. Results are represented as a scatter plot of pooled samples, with each dot as a pool of 5 samples at 24 and 48 h after immersion, using muscle as calibrator and comparing the expression with mock-immunized control. A significant modulation of MxA gene (*p* < 0.05) was observed at 24 h (Figure 2), as well as an increase at the same time for ISG12 gene (Figure 2), although not statistically significant due to the high individual variability.

**Figure 3:**
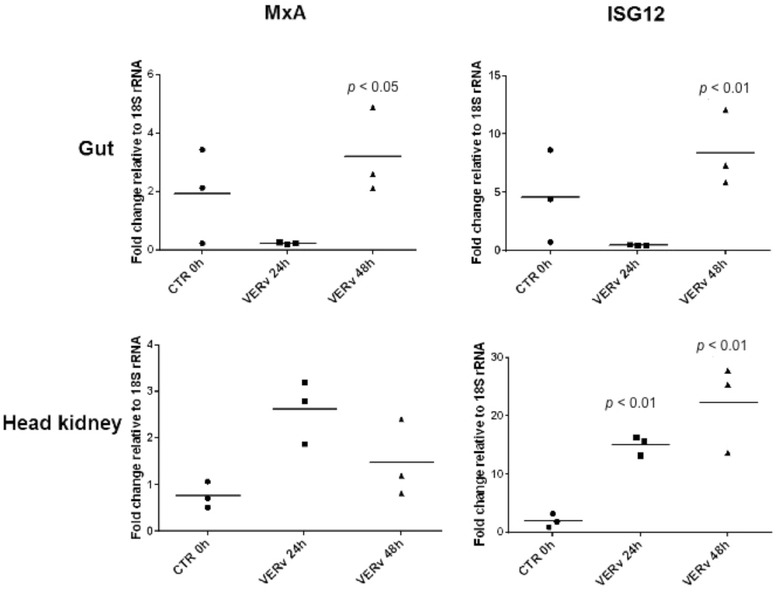
**Real-time quantitative PCR.** The expression level of genes coding for MxA and ISG12 in gut and head kidney samples are shown in control fish (CTR Group) and in fish immunized intraperitoneally with formalin-inactivated virus (VERv Group) at 24 and 48 h post immunization. The quantitative PCR amplification was performed in PCR arrays, and each point represents the mean ± SD of three single samples.

The expression of genes encoding for relevant antiviral genes in head kidney and gut after intraperitoneal immunization with formalin-inactivated VERv is shown in Figure 3 as a scatter plot of individual samples, with each dot representing single samples at 24 and 48 h after injection, and compared to the expression in control fish. Regarding the interferon-inducible MxA gene, a significant upregulation of MxA gene (*p* < 0.05) was observed at 48 h in the gut (Figure 3A), whereas in head kidney a not meaningful mean increase tendency was observed at 24 h (Figure 3C). A significant modulation of ISG12 gene (*p* < 0.05) was observed at 48 h in head kidney (Figure 3D), while in the gut a significant increase at 48 h (Figure 3B) and at 24 h (Figure 3D) was registered.

### Immunohistochemistry

**Figure 4:**
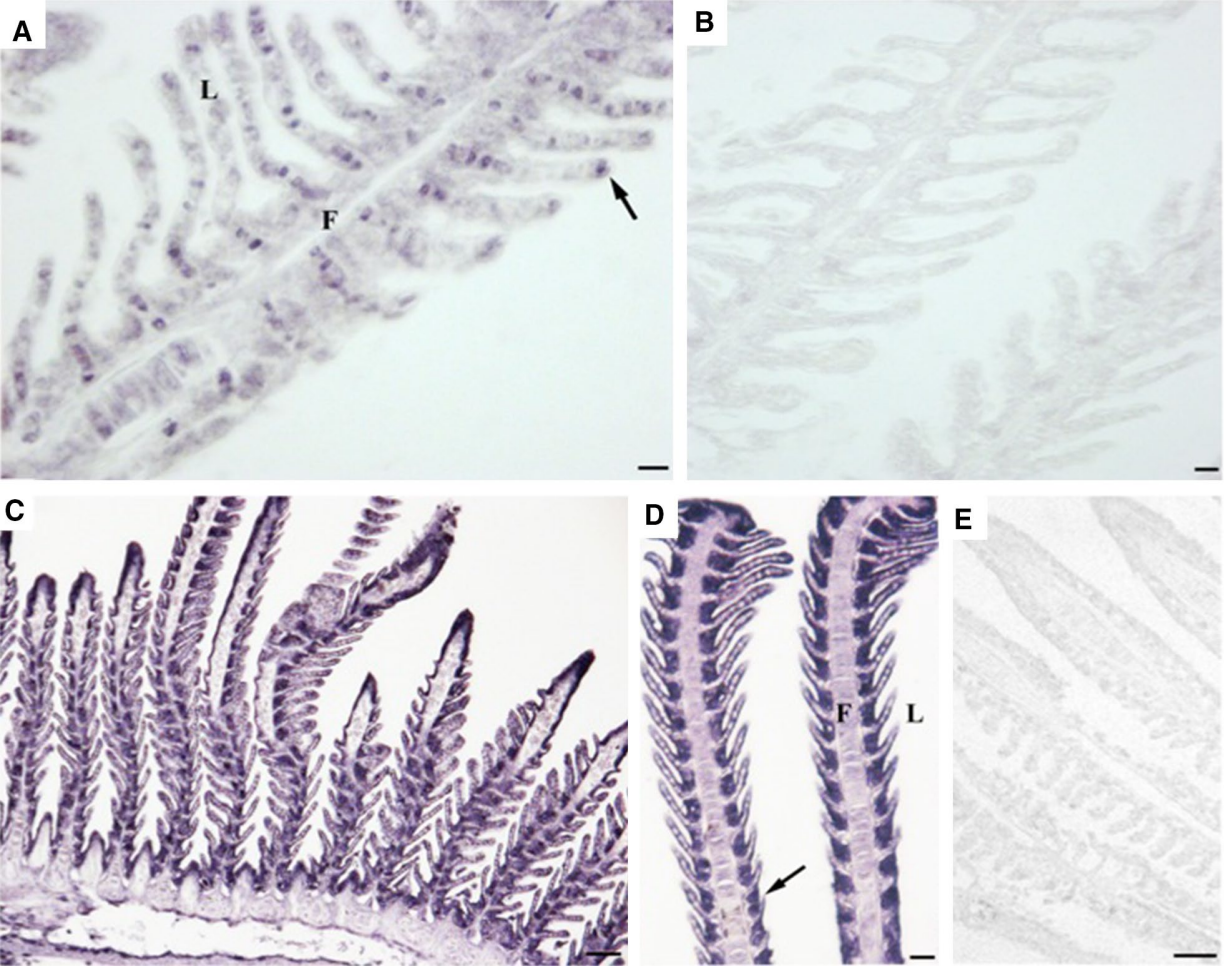
**Immunoreactive uptake in gills of formalin-inactivated VERv immersed sea bass with mAb 4C3 (A, B) and pAb 283 (C, D, E). A** Gill epithelium showing the presence of immunoreactivity employing mAb 4C3 (arrow). Scale bar = 10 μm. **B** Negative control showing the absence of immunoreactivity in gill tissue with mAb 4C3. Scale bar = 10 μm. **C** Gill epithelium showing the presence of immunoreactivity using pAb 283. Scale bar = 50 μm. **D** Higher magnification of immunoreactivity with pAb 283 (arrow). Scale bar = 20 μm. **E** Negative control showing the absence of immunoreactivity in gill tissue with pAb 283. Scale bar = 50 μm. F: filament, L: lamella.

Formalin-inactivated VERv was detected in the gills of sea bass at 48 h post-immersion by employing IHC. In particular, the mAb 4C3 (Figure 4A) recognised scattered cells, with a strong positive signal, distributed in the gill filaments and parallel secondary lamellae. In addition a weak reaction was homogeneously detected in the gill mucosal tissue. Notably, the control specimens (no immersion immunized animals) did not evidence any mAb 4C3 staining (Figure 4B). Differently, in immersion fish the pAb 283 (Figures 4C and D) confirmed the presence of inactivated VERv in the sea bass gills 48 h after the immersion, showing a strong reaction in the thin epithelium covering the secondary lamellae and in the basal cells localized between the secondary lamellae on gill filaments. No reaction was found in control sections without primary antibody (Figure 4E).

### Vaccine potency

**Figure 5:**
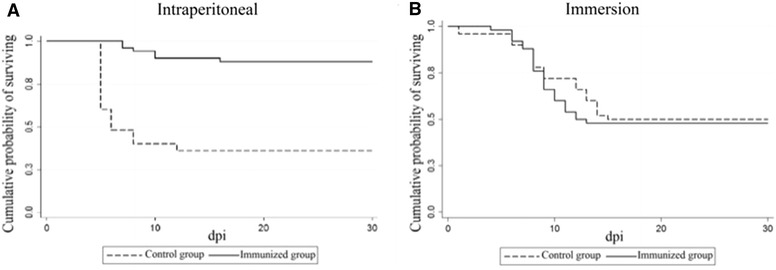
**Cumulative survival rate of challenged sea bass previously immunized either by intraperitoneal injection or by immersion with formalin-inactivated VERv.** Control group: fish mock-immunized intraperitoneally or by immersion with PBS 0.01 M; Immunized group: fish immunized (**A**) intraperitoneally or (**B**) by immersion with formalin-inactivated VERv; dpi: days post infection.

The challenge was performed only on formylated groups (i.p. and immersion immunized), as described above. Cumulative mortality was of 12% in the i.p. immunized group and of 64% (Figure 5A) in the positive controls, with significant differences between groups (*p* < 0.001). The RPS of the i.p. immunized group was 81.9%. On the other hand, immersion immunized fish showed a cumulative mortality of 50%, whereas in the positive control group it turned out to be of 52% (Figure 5B), with no differences between groups (*p* > 0.05) and a RPS of 1.6%.

## Discussion

*Betanodavirus* infection is characterized by a high pathogenicity and is associated with extensive mortality in fish farms [27]. Different attempts for immunization had previously been tested by inactivating the virus with either formalin [5, 28, 29] or binary ethylenimine (BEI) [28, 30], by the use of recombinant capsid proteins [31–33] or by DNA-based vaccines [33, 34]. Although some protection was obtained in sea bass fry by intramuscularly injecting a recombinant protein or synthetic peptides [13, 14], no vaccines are presently available to be used in *D. Labrax* (Additional file 1). In our study we tested different inactivation methods for *Betanodavirus*, namely by using formylation, BPL and temperature, with the aim of producing an effective antigen.

The inactivated antigen was then administered to fish through systemic (intraperitoneal) or mucosal (immersion) routes and serological results showed that formylation was the best inactivation way for the production of specific and neutralizing serum IgM after i.p. immunization. On the other hand, fish immunized i.p. with BPL and temperature-inactivated virus also showed the presence of specific serum IgM, but the high variability among individuals resulted in a poor statistical significance of data. This latter observation could be explained by assuming that the inactivation method may result in a loss of antigenicity, which in turn results in a decrease of efficient antigenic sites and, correspondingly, to the measured high standard variation in heat and BPL group. Indeed, several attempts were performed to decrease the concentration or the treatment time with formalin, BPL and temperature, all unsuccessful (data not shown) due to the resistance of *Betanodavirus* to chemical and physical inactivation [28, 35–38]. However, the inactivation protocols we suggested and reported in this work were able to completely inactivate the VERv.

The effectiveness of immunization with formalin-inactivated *Betanodavirus* administered i.p. was previously tested in other fish species. A significant specific antibody titer was measured in groupers (*E. septemfasciatus*) together with protection after challenge with live virus [5, 28]. Also, a single injection of formalin-inactivated *Betanodavirus* (RGNNV genotype) induced a humoral response and protection in Asian sea bass [29]. These data suggest that formylation can be a useful protocol for antigen preparation.

After having confirmed the presence of a humoral response induced by i.p. administration in sea bass, we examined the effects on the transcription of genes coding for antiviral responses. Interferon exerts its biological functions mainly through the downstream activation of Mx and ISG genes [39–42], which are both interferon-inducible and known to play an active-role in antiviral immune responses [43, 44]. Mx proteins are key players of antiviral responses, triggered by interferon type I (IFN-I) in response to viral infections and have been considered the main factor in determining the resistance of fish species to nodavirus infections. Consequently, Mx gene transcription can be used as an IFN-I system stimulation reporter [45]. Two different Mx genes had been identified in European sea bass, proving that the isoform MxA is particularly modulated in nodavirus-infected sea bass [20, 46]. As previously shown [47], the ISG12 gene can also be employed in sea bass as a marker of antiviral activities, since this ISG isoform is well expressed in gills [22], and significant ISG12 gene modulation can be induced in sea bass after in vitro stimulation with virus-mimicking substance poly I:C [47].

Our results showed that both MxA and ISG12 genes were significantly upregulated in head kidney and gut, thus indicating an effect of the formylated antigenic preparation. Based on promising serological and molecular results, we tested the efficacy of the immunization with formylated antigen given i.p. by challenging fish with live virus (RGNNV 283.2009). The results showed a high protection rate (81.9%) and, therefore, a single dose of i.p.-injected, formalin-inactivated RGNNV can be considered sufficient to elicit an in vivo protection of sea bass. The potential of our formalin-inactivated viral preparation as an effective immunogen was confirmed by: (1) a measurable humoral response; (2) a modulation in the expression of antiviral genes; (3) a seroconversion assay positivity; (4) a high protection after viral challenge.

Early life stages of *D. labrax* are the preferred target of *Betanodavirus*, reaching a mortality of 100% [35, 48] and in light of this we also investigated the possibility of immunizing sea bass at a young age through mucosal routes, namely by immersion. The efficacy of immersion immunization in eliciting humoral and cell-mediated immune responses has already been described is other fish species [49, 50]. Other interesting approaches have been explored by using inactivated VERv in immersion immunization, i.e. by employing nano-encapsulated formalin-inactivated or BEI-inactivated *Betanodavirus* preparations for the protection of grouper larvae (*Epinephelus coioides*) from NNV disease [28].

After analyzing by ELISA the results from immersion immunization with formylated nodavirus, we observed the presence of very low but still detectable specific serum IgM, which suggests that a single immersion may induce a weak humoral response in sea bass. The use of antigens encapsulated in nanoparticles could be useful to improve the delivery and the uptake also in sea bass, but this assumption needs further investigation. Furthermore, also the experimental groups immunized with BPL- and temperature-inactivated virus showed a scattered presence of specific serum IgM, resulting in a high individual variability and poor statistical significance of data, again attributable to a possible loss of antigenicity.

Being the gills directly involved in antigen uptake during immersion immunization, we analyzed the transcription of MxA and ISG12 genes at 24 and 48 h post immersion with formylated antigen, and we observed a significantly upregulation of these genes at 24 h, likely suggesting a possible antiviral response in the tissue. Following immersion immunization, the Mx and ISG12 transcription resulted much higher in the gills than in the kidney or gut after i.p. immunization. We can speculate that this difference could be due to the route of antigen uptake, because gills are in direct contact with the environment and must be armed with immune defences for the protection against pathogens [51]. Importantly, the presence of viral particles was demonstrated by IHC in the gill epithelium by using antibodies against the virus capsid protein (mAb 4C3) and against the whole virus (pAb 283) [25].

It should be noted that the infection with sub-optimal doses of live virus by immersion induced an effect, measured by the presence of significant antibody levels after 30 days (Figure 1) [15, 20], and this could raise the possibility of vaccinating fish with a live/attenuated virus, as already shown in a grouper species (*E. septemfasciatus*) [52, 53]. However, the use of live pathogens for fish immunization raises concerns about their containment and poses risks of vertical/horizontal transmission [27]. To confirm these concerns, we observed by RT-PCR the presence of VERv in brain samples from all fish infected by sub-optimal doses of live virus (data not shown).

In summary, data obtained from immersion-immunized fish, including the lack of protection in challenge trials, suggest that this way of administration needs to be further investigated also with studies including boosting/adjuvants.

## Additional file

10.1186/s13567-016-0376-3 **Summary of main characteristics and results obtained with experimental vaccines against VER.** The table describes the published works on attempts to produce a vaccine against VER, and corresponding references.

## Abbreviations

VER: viral encephalopathy and retinopathy
VNN: viral nervous necrosis
VERv: encephalopathy and retinopathy virus
RGNNV: redspotted grouper nervous necrosis virus
i.p.: intraperitoneal injection
BPL: β-Propiolactone
qPCR: real-time quantitative PCR
MEM-10: minimum essential medium
RPS: relative percent survival
MS-222: tricaine methanesulfonate
IHC: immunohistochemistry
pAb 283: policlonal antibody against Betanodavirus
mAb 4C3: monoclonal antibody against Betanodavirus capsid protein
BSA: bovine serum albumine
PBS: phosphate buffered saline
ELISA: enzyme-linked immunosorbent assay
SD: standard deviation
OD: optical density
FBS: fetal bovine serum
CPE: cytopathic effects
IgM: immunoglobulin M
IFN-I: interferon type I
