# Supplementary material for: A formalin-inactivated immunogen against viral encephalopathy and retinopathy (VER) disease in European sea bass (Dicentrarchus labrax): immunological and protection effects
Source: Vet Res. 2016 Sep 2;47(1):89. doi: 10.1186/s13567-016-0376-3 (PMC5010674; doi:10.1186/s13567-016-0376-3)
Supplement: Supplementary file 1 — 10.1186/s13567-016-0376-3 Summary of main characteristics and results obtained with experimental vaccines against VER. The table describes the published works on attempts to produce a vaccine against VER, and corresponding references. [file 13567_2016_376_MOESM1_ESM.docx]

| **Fish** | **Species** | **Vaccinal technology** | **Viral genotype used** | **Delivery method** | **Dose** | **Fish weight (g)** | **protection to challenge (RPS)** | **References** |
| --- | --- | --- | --- | --- | --- | --- | --- | --- |
|  |  |  |  |  |  |  |  |  |
| European sea bass | *D.labrax* | different synthetic peptides | DIEV | i.m. | 0.2 mg | 20-25 | 4-65% (mortality) | [13] |
|  | *D. labrax* | recombinant capsid protein | MGNNV | i.m. | 100 and from 20 to 0.5 μg | 22-66 | 27.4-88.9 | [14] |
| Asian sea bass | *L. calcarifer* | Inact. VNNV | RGNNV | i.m. | 10^8^ TCID_50_ | 5.5 | n.d. | [54] |
|  | *L. calcarifer* | recombinant capsid protein | n.d. | i.m. | 50 μg | 10-15 | 76 | [55] |
|  | *L. calcarifer* | DNA capsid + nanoparticles | n.d. | oral | n.d. | 10-16 | 60 | [34] |
| Halibut | *H. hippoglossus* | recombinant capsid protein | SJNNV | i.p. | 100 μg | 695 | n.d. | [31] |
|  | *H. hippoglossus* | recombinant capsid protein | AHNV | i.p. | 10 e 50 μg | 25 | n.d. | [56] |
| Turbot | *S. maximus* | recombinant capsid protein | SJNNV | i.p. | 100 μg | 1.3 | 33-82 | [31] |
|  | *S. maximus* | recombinant capsid protein | AHNV | i.p. | 50-10 μg | 2.2 | 17-67 | [33] |
|  | *S. maximus* | DNA capsid | AHNV | i.m. | 10 μg | 2.2 | no protection | [33] |
| Grouper | *E. septemfasciatus* | Inact. VNNV | RGNNV | i.p. | 10^9^ TCID_50_ | 12.3 | 67-100 | [5] |
|  | *E.coioides* | Inact. VNNV | HGNNV | i.m. | 10^7^ TCDI_50_ | 0.2-2 | 39-79 | [28] |
|  | *E.coioides* | Inact. VNNV | HGNNV | bath | 10^5^-10^6^ and 10^7^ TCID_50_ | 0.2-2 | 75-88 | [28] |
|  | *E. septemfasciatus* | Inact. VNNV | RGNNV | i.p. | 10^7.3^ TCID_50_ | 75 | >60 | [7] |
|  | *E. septemfasciatus* | Inact. VNNV | RGNNV | i.m. and i.p | from 10^8.5^ to 10^6.5^ TCID_50_ | 25.3 | 82-94 | [7] |
|  | *E. fuscoguttatus* | Inact. VNNV | RGNNV | i.p. | 10^10.5^ TCID_50_ | 5 | 86-100 | [54] |
|  | *E. coioides* and *E. tukula* | Inact. VNNV | HGNNV | i.m. and i.p | 10^8^ and 10^9^ TCID_50_ | 1 and 45Kg | no challenge | [57] |
|  | *E. coioides* | Inact. VNNV | RGNNV | bath | 5 × 10^5^ TCID_50_ | 0.18 | no challenge | [58] |
|  | *E. coioides* | Inact. VNNV in A. salina | RGNNV | oral | n.d. | 0.18 | no challenge | [58] |
|  | *E. septemfasciatus* | recombinant capsid protein | RGNNV | i.m. | 60 | 28 | 35–88 | [59] |
|  | *C. altivelis* | recombinant capsid protein | RGNNV | i.m. | 0.21 mg | 27 | 67.5 | [60] |
|  | *E. coioides* | recombinant capsid protein in A. salina | n.d. | oral | n.d. | 8-18-19 dph | 80-86 | [32] |
|  | *E. septemfasciatus* | live NNV | RGNNV | bath | 10^5.3^ TCID_50_ | 35-119 | 92-95 | [53] |
|  | *E. septemfasciatus* | live NNV | RGNNV | i.m. | 4.3 TCID_50_ | 35-119 | 88-100 | [17] |
|  | *E. septemfasciatus* | live NNV | RGNNV | i.m. | 10^4.3^ TCID_50_ | 53.7 | 95.8 | [61] |
|  | *E. lanceolatus* | VLP | DGNNV | i.m. | 10. 100. 250 μg | 20 | n.d. | [62] |
|  | *E coioides* | VLP | OGNNV | i.m. | 1.5 or 15 μg | 0.36 | n.d. | [63] |
|  | *E. septemfasciatus* | VLP | RGNNV | i.p. | 50 μg | 71.5 | 100 | [64] |
|  | *E. septemfasciatus* | VLP | RGNNV | oral | 50 μg | 71.5 | 57 | [64] |

Legend: i.m.: intramuscular injection; i.p.: intraperiotenal injection; n.d.: not determined; dph: days post hatchery; VLP: virus like particles.
